# Use of memory strategies among younger and older adults: Results from objective and subjective measures

**DOI:** 10.1590/S1980-57642011DN05020006

**Published:** 2011

**Authors:** Aline Teixeira Fabricio, Mônica Sanches Yassuda

**Affiliations:** 1 Undergraduate in Gerontology, Escola de Artes, Ciências e Humanidades da Universidade de São Paulo, São Paulo SP, Brazil;; 2 Associate Professor in Gerontology, Escola de Artes, Ciências e Humanidades da Universidade de São Paulo, São Paulo SP, Brazil.

**Keywords:** memory, aging, youth, strategies

## Abstract

Memory plays a fundamental role in the identity of people and in human life, as it enables us to interpret our surroundings and make decisions. It is known that the aging process can be accompanied by cognitive decline in some memory sub systems. However, the use of memory strategies can help encoding and retrieval of new information.

**Objective:**

The aim of this study was to identify and compare, using objective and subjective measures, which recall strategies are used spontaneously by young and older adults.

**Methods:**

Twenty-six first-year college students, and thirty-three seniors enrolled at the Third Age University of the same campus, completed a visual memory test including 18 black and white pictures, memorized a short story, and completed an open question about memory strategies, a memory check list to indicate strategies used, and a memory self-efficacy scale. The Bousfield categorization measure was also calculated from the recall protocol.

**Results:**

Young adults demonstrated better performance than the older adults on the memory tasks, and were also more confident. Both groups reported using similar strategies.

**Conclusion:**

Young and older adults seem to tackle memory tasks in similar ways but young adults outperform seniors.

## Abstract (pt)

A memória tem um papel fundamental na identidade das pessoas e um papel vital na vida humana, pois nos permite interpretar o nosso meio e tomar decisões. É sabido que o processo de envelhecimento pode ser acompanhado pelo declínio cognitivo em alguns subsistemas de memória. No entanto, o uso de estratégias de memória pode auxiliar na codificação e recuperação de novas informações.

**Objetivo:**

Identificar e comparar, por meio de medidas objetivas e subjetivas, as estratégias de memória utilizadas de forma espontânea por jovens e idosos.

**Métodos:**

Vinte e seis estudantes universitários do primeiro ano e trinta e três idosos matriculados na Universidade Aberta à Terceira Idade completaram um teste de memória visual, incluindo 18 figuras em preto e branco, memorizaram uma história curta, completaram uma questão aberta sobre o uso de estratégias de memória, e selecionaram as estratégias que usaram dentre uma lista de possíveis estratégias, e preencheram uma medida de autoeficácia para memória. A medida de categorização de Bousfield foi calculada a partir do protocolo de recordação da lista de figuras.

**Resultados:**

Os jovens apresentaram desempenho melhor do que os idosos nas tarefas de memória, e mostraram-se mais confiantes. Os dois grupos relataram usar estratégias semelhantes.

**Conclusão:**

Os jovens e idosos parecem enfrentar as tarefas de memória de forma semelhante, no entanto, os jovens superam os adultos idosos no desempenho objetivo.

Over recent years, the Brazilian population has been undergoing a process of demographic transition, characterized by a rising proportion of older adults in the general population. The aging process can be accompanied by decline in physical and cognitive abilities which are modulated by life style.^1^ Studies have reported that during aging, many elderly experience changes in cognitive functions, especially in memory.^2^

Memory plays a fundamental role in the identity of people and in human life, as it enables us to interpret our surroundings and make decisions. It can be defined as the capacity that some living organisms have to encode, store and retrieve information.^3,4^

Memory is composed by several sub-systems. According to Gazzaniga and Heatherton,^3^ when time is used as criterion, memory could be classified as sensory memory, short-term memory (STM) and long-term memory (LTM). Salthouse^2^ summarized recently that the typical cognitive profile of healthy older adults entails early linear decline in sub systems dependent upon on line processing and new learning, such as working memory (STM) and episodic memory (LTM), yet, it entails stability in sub systems which depend on accumulative learning processes, such as semantic memory (LTM). Studies have also suggested minor changes in implicit memory tasks (LTM).

Therefore, the cognitive aging literature suggests that in the absence of compensatory mechanisms, such as the use of memory strategies or contextual cues during encoding, most older adults will show worse performance when compared to younger individuals, particularly in episodic memory tasks. Nevertheless, performance differences between young and old adults may be reduced by adjusting learning conditions, such as offering longer study time for seniors or encouraging them to use memory strategies.

Although largely studied in other countries, cognitive training has received limited attention in Brazil. Carvalho et al.^5^ reported that older adults have the potential to improve memorization and access to information through the use of memory strategies, such as categorization. Memory strategies include methods or techniques that positively impact memory performance, as they help organize new information or highlight meaning or relevance.^6^ Strategies may be external, such as written reminders, calendars and diaries, or internal, such as creating mental images, categorization, verbal association, the method of loci, and the face-name method.^7^

According to West,^7^ young and old adults do not differ on the type of memory strategies employed. Both groups make use of external strategies for most situations. Young adults, however, employ strategies more spontaneously and more frequently than elderly individuals. In addition, older adults may be discouraged from using strategies due to negative beliefs regarding their capacity to improve memory performance.^8^

The aim of the present study was to compare younger and older adults, with similar educational level on two episodic memory tasks and on objective and subjective measures of strategy use. A measure of memory self-efficacy, and was also included in the study protocol in order to evaluate differences between age groups on subjective memory.

## Methods

### Participants

A total of 26 young individuals in their first year at the University of São Paulo, and 33 mature and older adults participating in the University of Third Age were interviewed during the second semester of 2009, and the first semester of 2010.

The young participants were 17 years and older (17-34 years), while mature and older adults were 50 years and older (50-81 years). All participants of both age groups had studied to high school level, i.e. they had at least eleven years of schooling. The sample characteristics are shown in Table 1. The older group included a few individuals who had completed high school in condensed programs (n=7).

**Table 1:** Mean and standard deviation for sociodemographic characteristics of participants.

	Young (n=26)	Elderly (n=33)	p value
Age	20.04 (3.36)	66.27 (18.44)	0.000*
Schooling	12.31 (0.88)	13.12 (2.13)	0.680*
Income			
From 0 to 5 MW (%)	57.6	69.6	0.299**
From 5 to 10 MW (%)	30.7	12.1	
Above 10 MW (%)	7.6	18.1	
Gender (No. of women)	19	20	0.315**

* p value refers to Student's t test for independent samples;

** Chi-square test; MW: minimum wage.

### Instruments

The following instruments were applied:

[1] A sociodemographic questionnaire including questions on sex, age, schooling, and income;
[2] Geriatric Depression Scale with 15 questions (GDS);^2^
[3] Adapted Memory Self-efficacy Questionnaire^9,10^ (in the adapted version, participants are asked to mark the number of items they believe they are able to memorize on different memory tasks, as opposed to marking their level of confidence for memory tasks of varying degrees of difficulty);
[4] 18 Picture Test,^11^ where the pictures can be categorized into three semantic groups;
[5] Story sub-test of the Rivermead Behavioral Memory Test, with 21 ideas, version B (RBMT);^12^
[6] Two open questions on the use of strategies, “What did you do to recall the pictures?” and “What did you do to recall the story?”,
[7] Checklist of possible strategies, where participants had to choose the strategies used after memorizing the pictures and story.

In order to analyze the answers to the open questions on strategy use, strategy categories were created based in the responses given by the participants. The use of strategies was also analyzed based on the Bousfield categorization measure,^13^ frequently used in studies on memory strategies.^14^ This measure is used to objectively quantify the degree of semantic organization of the recall of the pictures on a scale of 0 to 1, where 0 represents no categorization and 1 represents total categorization, i.e. values closer to 1 indicate a higher degree of grouping by semantic category of the items recalled. The index is calculated by the rate of intra-categorical repetition based on the formula [RR=r/n-1], where r represents the number of items of the same category recalled together, and n denotes total items recalled.

### Procedures

The older adults were invited to take part in the study while they were attending activities. The young individuals were invited to take part in the study during classes at the same university. The assessments were conducted in age segregated groups of five to 12 participants.

For the 18 Picture Test, participants studied the pictures for one minute. Next, they completed the Digit Symbol WAIS-III sub-test as an interference task for 2 minutes and then wrote on a blank sheet of paper the names of the pictures they could recall. After they completed the memory task, they answered the questions about strategy use. For the RBMT story, participants read the story for three minutes, completed the Clock Drawing Test and then wrote the information they could recall about the story on a blank sheet of paper. After they completed the memory task, they answered the questions about strategy use. All participants were supervised by the team of researchers to ensure that all had exactly the same time to carry out the tasks, and that answers were not voiced aloud. This project was approved by the Research Ethics Committee of the Psychology Institute of the University of São Paulo (No. 2010.035).

### Data analysis

In the analyses, age (young-old) was the main independent variable of interest. Education, income, sex and GDS scores were used to describe the sample. The dependent variables were the scores for the 18 Picture test, the RBMT story, Memory Self-efficacy Questionnaire, and the objective and subjective strategy use measures. The results from the younger and older participants were compared using Student’s t test for independent samples based on normal data distribution for the following variables: GDS, 18 Picture Test, RBMT story, and Memory Self-efficacy Questionnaire. Mann-Whitney’s U-test was used to identify differences between the groups for the variables age, schooling, Bousfield measure, as they did not follow normal distribution. The Chi-square test was used to compare categorical variables such as income and gender. The level of significance adopted was 5% (0.05), i.e. p<0.05. The data were entered twice into version 3.1 of the Epidata Program. The SPSS v.17.0 software package was employed for the statistical analysis.

## Results

Table 1 shows the sociodemographic profile for young and elderly participants. No significant difference was found between the groups for schooling, income and sex.

Table 2 contains data for the cognitive variables, the Bousfield measure, and GDS scores. The analysis revealed a significant difference between the two groups for the GDS (p=0.035), with young participants reporting a greater number of depressive symptoms. Young participants achieved significantly higher scores on all cognitive scales and had higher self-efficacy for memory tasks. No difference in the Bousfield categorization measure was found between the groups. Both young and older adult groups organized the 18 figures in a similar manner to facilitate recall.

**Table 2:** Means and standard deviations for cognitive variables, strategies, depressive symptoms, and self-efficacy measures.

	Young	Elderly	p value
GDS	3.15 (1.46)	2.31(1.49)	0.035*
Pictures	14.08 (1.98)	12.64 (2.45)	0.018*
Stories	16.00 (3.75)	12.52 (2.73)	0.001*
Self-efficacy	26.76 (3.69)	23.96 (3.44)	0.004*
Bousfield	0.64 (0.23)	0.78 (0.68)	0.663**

* p value refers to t test for independent samples;

** p value refers to the Mann-Whitney U test for independent samples.

The memory strategies were analyzed using closed and open questions (Tables 3, 4, and 5). The number of strategies cited during recall of the pictures and stories in open questions were tallied separately, where participants wrote descriptions of what they did to recall items. No significant difference in the number of strategies reported by young and elderly was found, with a total of 12 different types of strategy listed. Categorization was the most cited strategy in both groups. In total, 17 younger individuals and 17 older adults reported using this strategy. These data are shown in Table 3.

**Table 3:** Spontaneous strategies reported for picture recall.

Strategies cited	Young (n=26)	Elderly (n=33)
Categorization	17	17
Memorized order (top to bottom, as pictures appear)	3	2
Effort/try/memorize/ read several times	3	6
Association (with clothes, taste, personal objects)	2	5
Mental Image (short stories)	3	1
Repetitions	1	1
Remembered the first letter of Pictures	0	1
Photographic memory	1	0
Attention	0	1
Dynamic reading	0	1
Counted total number of pictures	0	1
Nothing	0	1

**Table 4:** Spontaneous strategies reported for story recall.

	Young (n=26)	Elderly (n=33)
Effort/try/memorize/ read several times	7	6
Mental Image (short stories)	2	1
Association (with news, events)	1	3
Underlining	1	1
Key words (firemen, Holland, names of places)	4	4
Chronological order of story (order of events)	3	3
Assimilated content (use understanding, interpretation)	2	0
Breakdown story into blocks	1	2
Pay attention to/memorize key points	4	4
Mentally retell	2	1
Repetition	1	0
Categorization	0	1
Words at beginning of each paragraph	0	1
Nothing	3	6

**Table 5:** Number of strategies reported during picture and story recall on closed questions (mean and standard deviation in brackets).

	Young	Elderly	p value
Strategies for pictures	3.15 (1.18)	3.06 (0.94)	0.74
Strategies for stories	1.84 (1.10)	2.26 (1.21)	0.21

The most cited strategy for story recall was “effort/memory/read several times”. No significant difference in use of strategies to memorize stories was observed between young and elderly individuals. A total of 15 different strategies were cited. On this task, a greater number of individuals reported not employing any method to aid recall of the story - 3 young individuals and 6 older adults. The data is shown in Table 4.

No significant difference was found between the age groups for the number of strategies used.

When participants chose the strategies used to recall pictures and stories in closed questions, both young and elderly participants chose the following strategies: use of mental image, organized figures in groups (categorization), and association. In the recall of the stories, both groups selected use of mental image and association as the most used techniques (Table 6).

**Table 6:** Strategies for picture and story recall on closed questions

	Young (n=26)	Elderly (n=33)
Pictures		
Mental image	21	25
Organized pictures into groups	22	25
Association	17	17
Remembered the first letter of pictures	1	5
Remembered objects in pocket	3	8
Counted total number of pictures	7	11
Concentrated on order pictures appeared	9	5
Stories		
Created a mental image	18	15
Association	15	14
Remembered things that had happened to you or had seen on TV	7	9

## Discussion

The use of memory strategies can enhance performance on memory tasks as they act as mediators at the time of encoding and recall. The aim of the present study was to compare the use of memory strategies by young and elderly individuals, and to identify which strategies are spontaneously most used by the two groups. In addition, performance on memory tasks was also compared. Results suggested that categorization and association were the strategies most frequently cited. Many of the participants reported making an effort, reading several times and memorizing. These unspecific techniques were predominantly used for recalling the stories.

Despite the superior performance of young individuals on memory tasks, no significant difference between the two groups was found for level of categorization, assessed with the Bousfield measure, in the recall of the 18 pictures. In the open question about strategy use, the number and type of strategies cited were similar for both age groups. This finding seems to indicate that, when matched for schooling and income, young and elderly participants perform memory tasks in a similar manner. Therefore, the data from the present study confirmed the findings in the literature, indicating superior performance among young individuals on memory tests despite employing similar strategies.^8^

Connor^15^ reported that older adults use memory techniques in a less effective way compared to younger individuals, but benefit from direct or indirect instructions on the use of new memory strategies. Werlang et al.,^16^ in a study on memory strategies for correct use of medications in older adults, reported that the use of strategies was able to minimize and compensate for mnemonic difficulties, contributing to greater autonomy and quality of life. Pinto^17^ reported that the use of memory strategies can attenuate the discrepancy in performance between young and elderly subjects. The author showed that practice using strategies can reduce the processing overload and improve performance.

Freire et al.^18^ examined the effects of strategy use for contextual memory tasks in young and elderly individuals. These authors proposed that deficits in contextual memory in older individuals can be reversed with the use of strategies during encoding, and suggested that for young participants strategy use did not bring performance change.

In the present study, memory self-efficacy was lower for the older group than that reported by the young individuals. However, this does not appear to have influenced effort on memory tasks, as strategy use was similar between the age groups. These results mirror those found in previous studies.^9,10^

It is interesting to note that the young participants in the present study had higher scores on the GDS compared to the elderly individuals. Although the instrument used has been devised for use among older individuals, this finding corroborates results from an epidemiological study which indicated that the presence of depression was more common among young individuals.^19^

Limitations of this study include the fact that results can be associated to the characteristics of the sample. GDS scores, for instance, may be affected by the fact that seniors were involved in the University of Third Age. In addition, the sample was composed of mature and young seniors and all of them had at least 11 years of schooling. Therefore, findings may not be generalized for samples with different characteristics.

Results allow us to conclude that there was no difference in the spontaneous use of memory strategies between the younger and older groups. Both devised similar strategies, yet, the performance of young subjects was superior. Also, the young individuals were found to be more confident about memory performance. These data are in line with the hypothesis that age differences in memory performance may be explained by variables other than strategy use and they may stem from age associated neurobiological changes.
